# Less is more: Fewer attract-and-kill sites improve the male annihilation technique against *Bactrocera dorsalis* (Diptera: Tephritidae)

**DOI:** 10.1371/journal.pone.0300866

**Published:** 2024-03-21

**Authors:** Thomas Fezza, Todd E. Shelly, Abbie Fox, Kyle Beucke, Eric Rohrig, Charlotte Aldebron, Nicholas C. Manoukis

**Affiliations:** 1 United States Department of Agriculture, Animal Plant Health Inspection Service, Waimanalo, Hawaii, United States of America; 2 United States Department of Agriculture, Agricultural Research Service, Daniel K. Inouye US Pacific Basin Agricultural Research Center, Hilo, Hawaii, United States of America; 3 United States Department of Agriculture, Animal Plant Health Inspection Service, Plant Protection and Quarantine, Palmetto, Florida, United States of America; 4 California Department of Food and Agriculture, Sacramento, California, United States of America; 5 Division of Plant Industry, Florida Department of Agriculture and Consumer Services, Gainesville, Florida, United States of America; Bayero University Kano, NIGERIA

## Abstract

The Male Annihilation Technique (also termed the Male Attraction Technique; “MAT”) is often used to eradicate pestiferous tephritid fruit flies, such as *Bactrocera dorsalis* (Hendel). MAT involves the application of male-specific attractants combined with an insecticide in spots or stations across an area to reduce the male population to such a low level that suppression or eradication is achieved. Currently, implementations of MAT in California and Florida targeting *B*. *dorsalis* utilize the male attractant methyl eugenol (ME) accompanied with a toxicant, such as spinosad, mixed into a waxy, inert emulsion STATIC ME (termed here “SPLAT-MAT-ME”). While highly effective against ME-responding species, such applications are expensive owing largely to the high cost of the carrier matrix and labor for application. Until recently the accepted protocol called for the application of approximately 230 SPLAT-MAT-ME spots per km^2^; however, findings from Hawaii suggest a lower density may be more effective. The present study adopted the methods of that earlier work and estimated kill rates of released *B*. *dorsalis* under varying spot densities in areas of California and Florida that have had recent incursions of this invasive species. Specifically, we directly compared trap captures of sterilized marked *B*. *dorsalis* males released in different plots under three experimental SPLAT-MAT-ME densities (50, 110, and 230 per km^2^) in Huntington Beach, CA; Anaheim, CA; and Sarasota-Bradenton, FL. The plots with a density of 110 sites per km^2^ had a significantly higher recapture proportion than plots with 50 or 230 sites per km^2^. This result suggests that large amounts of male attractant may reduce the ability of males to locate the source of the odor, thus lowering kill rates and the effectiveness of eradication efforts. Eradication programs would directly benefit from reduced costs and improved eradication effectiveness by reducing the application density of SPLAT-MAT-ME.

## Introduction

The true fruit flies (Diptera: Tephritidae) comprise over 4,000 species, approximately 250 of which are serious agricultural pests of fleshy fruits and vegetables [1]. Established tephritid pests are commonly controlled through an Integrated Pest Management (IPM) approach that may incorporate mass trapping, application of synthetic insecticides and protein bait sprays, the release of natural enemies, the sterile insect technique (SIT), and the male annihilation technique (MAT) [2–4]. Despite improved management and regulatory strategies over the last century, the broad host range and high dispersal capability of many fruit flies along with increased global transport of people and goods have amplified the invasion threat of pest tephritids [5, 6].

Within the United States most invasions of exotic fruit flies occur in southern states, such as Texas, Florida, and southern California. MAT is a key tool for eliminating establishment of some species, particularly methyl eugenol-responding *Bactrocera* species, such as the oriental fruit fly, *Bactrocera dorsalis* (Hendel). This polyphagous species has been detected with increasing frequency in southern California [7] where it has been eradicated repeatedly [8]. This species was also the subject of a successful eradication effort in South Florida in 2015–2016 [9].

Operationally, MAT involves the application of male-specific attractants (in combination with an insecticide) at high densities in the environment to attract and kill males until the population has been reduced to such a low level that suppression or eradication is achieved. In several instances, MAT programs have successfully eradicated *B*. *dorsalis* using large numbers of fiberboards, coconut husks, or cotton rope impregnated with methyl eugenol and the toxicant naled deployed via aircraft or ground placement [10–12]. In addition to varying types of ispensers, there have been substantial differences in the amount of ME applied to individual dispensers as well as the density at which they were distributed in the area treated. For example, a survey of past MAT programs revealed that the amount of ME applied to dispensers varied from 8–23 g, with between 85 and 400 dispensers per km^2^ [12, 13].

In California, MAT has been implemented frequently against *B*. *dorsalis*. In 2019, there were six separate *B*. *dorsalis* eradication projects in Los Angeles, Sacramento, San Joaquin, Santa Clara, and Ventura counties. These projects are more frequent in southern California, especially the Los Angeles area. In California, MAT involves squirting a mix of lure, toxicant, and waxy carrier onto available surfaces instead of individual physical dispensers. In Florida, MAT was an essential tool against a large recent incursion [9] and continues to be used during eradication programs, applied as spot treatments (5–10 ml/spot) on roadside trees or utility poles. To minimize human health risks in the immediate outbreak area, MAT programs against *B*. *dorsalis* in both states utilize spinosad, a reduced risk insecticide with low mammalian toxicity [14, 15].

While highly effective, MAT as practiced in California and Florida is expensive owing largely to the high cost of the carrier matrix as well as labor. The carrier matrix, a waxy, inert emulsion termed SPLAT (ISCA Technologies, Riverside, CA), is a proprietary product that not only adheres to surfaces, but also serves as a controlled release matrix, an important feature given ME’s high volatility [16, 17]. For convenience, the SPLAT, ME and spinosad mixture applied in the field is termed SPLAT-MAT-ME. At the time of this study, the accepted protocol in California and Florida called for the application of approximately 230 SPLAT-MAT-ME spots per km^2^ (600 spots per mi^2^), which has been the standard for several decades.

To determine whether eradication might be achieved at a lower spot density, Manoukis et al. [13] compared the effectiveness of the standard density with higher (440 spots per km^2^) and lower (110 spots per km^2^) densities in a mark-release-recapture study conducted in a Hawaii macadamia nut orchard. The results showed that an application rate of 110 spots/km^2^ produced the greatest control, suggesting that use of a lower SPLAT-MAT-ME spot density might reduce costs while increasing efficacy, in agreement with two previous studies [18, 19]. However, all three of these studies were conducted in rural Keaau, Hawaii under often cloudy, cool, and wet conditions. As differences in humidity, temperature, wind, and vegetation could affect lure release and dispersal as well as the response of *B*. *dorsalis* males, the applicability of these Hawaii results to California and Florida was uncertain [20–22]. Furthermore, experiments in Hawaii were conducted with highly uniform applications of MAT, mirroring the orderliness of tree rows within a plantation. Such uniformity is absent in urban environments, where operational considerations and rules limit acceptable application sites.

The goal of the present study was to test the hypothesis that a lower density of SPLAT-MAT-ME may be more effective at attracting and killing *B*. *dorsalis* males than the higher densities currently used in eradication programs. Manoukis et al. [19] and Jang et al. [18] suggested that a higher density of ME-baited traps create olfactory interference, which may inhibit the ability of *B*. *dorsalis* males to locate traps resulting in fewer captures compared to a lower density of traps. Accordingly, adopting the same experimental framework as Manoukis et al. [13], the present study directly compares captures of sterile marked *B*. *dorsalis* males released within three different experimental SPLAT-MAT-ME site densities, i.e., 55, 110, and 220 per km^2^ in residential neighborhoods in southern California and the west coast of Florida, where *B*. *dorsalis* invasions have recently occurred.

## Material and methods

### Study sites

A total of six plots were established in California, three in the city of Anaheim and three in Huntington Beach. In Florida, six plots were selected in the Sarasota-Bradenton area. Each plot had an area of 0.65 km^2^ and a minimum distance of 1 km between plots (plot locations are provided in Table 1). Plots were bordered by multilane roadways and were in residential neighborhoods that did not include large, paved areas, such as malls and parking lots. The selected neighborhoods contained predominantly single dwelling homes with an average lot size of approximately 550 m^2^ and were in areas where *B*. *dorsalis* incursions have occurred in the past.

**Table 1:** Locations of experimental plots. Data are given as “longitude; latitude” in degrees. Each plot had an area of approximately 0.65 km^2^ with four distinct corners and a release transect that was a minimum of 100m from SPLAT-MAT-ME sites. Further data, including SPLAT point locations and release transect coordinates are given in (for full dataset of application locations please see [23]).

Location	Spots/km^2^	Corner 1 (NW)	Corner 2 (NE)	Corner 3 (SE)	Corner 4 (SW)
Anaheim, California	230	-117.95875572; 33.803073285	-117.94915411; 33.803074362	-117.94965523; 33.79637110	-117.95860139; 33.795932924
	110	-117.9325432; 33.79598398	-117.92398617; 33.796002583	-117.9237811; 33.788865138	-117.93248413; 33.788810815
	50	-117.94961143; 33.793151698	-117.94140837; 33.793081012	-117.94123481; 33.785531630	-117.94986351; 33.785473915
Huntington Beach, California	230	-117.98027105; 33.694043341	-117.97152760; 33.694007568	-117.97145880; 33.686814620	-117.98009344; 33.686793162
	110	-117.98350832; 33.672261983	-117.97505417; 33.672218742	-117.97534224; 33.665017383	-117.98418359; 33.664928044
	50	-117.96726118; 33.694045214	-117.95896439; 33.694119756	-117.95900071; 33.686827980	-117.9670798; 33.686795636
Sarasota-Bradenton, Florida 1	230	-82.541334427; 27.363416746	-82.5308668; 27.363317493	-82.53066848; 27.355885820	-82.54015275; 27.356140177
	110	-82.554678461; 27.440145440	-82.546797344; 27.440168805	-82.546752890; 27.432990424	-82.555244551; 27.433052741
	50	-82.587626537; 27.447748031	-82.579508356; 27.447671247	-82.579632716; 27.44034679	-82.587641942; 27.44042291
Sarasota-Bradenton, Florida 2	230	-82.55100000; 27.498999999	-82.543000017; 27.499999998	-82.543000000; 27.490999999	-82.549999999; 27.491999999
	110	-82.587626536; 27.447748031	-82.579508356; 27.447671247	-82.579632716; 27.44034679	-82.587641942; 27.44042291
	50	-82.644999999; 27.519000000	-82.63738167; 27.518421857	-82.637945340; 27.515011594	-82.6379453398; 27.515011594

Common vegetation within the California and Florida plots included palms, various varieties of citrus, other fruit fly hosts, along with various ornamental trees, hedges and shrubs. Releases were performed in California in August and September 2021 under an average daily temperature of 27.2°C, with a high of 34.9°C and low of 20.5°C, while experiments in Florida occurred in July and November 2022 with an average daily temperature of 25.2°C, with a high of 32.7°C and low of 17.7°C. No heavy rain occurred during any release-recapture interval. Weather data were obtained from NOAA-operated weather stations at John Wayne Airport in Orange County, CA and Sarasota-Bradenton International Airport in Sarasota, FL.

### Insects

*Bactrocera dorsalis* were obtained from a bisexual colony maintained at the USDA-ARS Daniel K. Inouye Pacific Basin Agricultural Research Center in Hilo, HI. This strain has been mass-reared since 1991 (approximately 350 generations) following a standard rearing protocol [24] and is housed in a building devoted exclusively to rearing that is maintained at 25 ± 1°C, 55 ± 3% rh, and a 12:12 L:D photoperiod.

The following procedures were adopted to maximize synchrony in immature development and thereby obtain batches of same-aged pupae for irradiation. Eggs (12 mL) were collected from colony cages and 0.5 mL aliquots were placed in 24 individual plastic containers (22 x 15 x 5 cm), each with 315 g of artificial diet (59.3% water, 29.9% wheat mill feed, 7.1% granulated white sugar, 3.4% torula yeast, 0.1% nipagen, and 0.1% sodium benzoate). These containers were placed in a larger fiberglass bin 50.0 x 32.0 x 15.2 cm (Plexton Stack-N-Nest, Lewis Systems, Watertown, WI, USA) held in a room devoted to larval production that is maintained at 26.5 ± 1.2°C, 62 ± 4% rh, and a 12:12 photoperiod. After 6 d, tap water was added to the fiberglass bins (2 cm depth), and the bins were relocated to a 16°C room for 18 h. During this time, the larvae emerged from the diet and into the 16°C water, which temporarily paused their development, thus allowing larvae of similar age to be collected. After the 18 h period, the water was drained, and larvae were placed on sterile corn cob pieces with a grain size of 2 mm and stored at 26.5 ± 1.2°C, 62 ± 4% rh, and a 0:24 photoperiod. Pupae were collected 48 h later and then held at 25 ± 1°C, 55 ± 3% rh, and a 12:12 photoperiod until irradiation. Under the conditions used, the duration of the pupal stage was 8 d.

### Marking protocol

*Bactrocera dorsalis* pupae were marked using fluorescent dye following established protocol [25] that is widely used in SIT programs [26]. Upon eclosion, adult flies often retain dye particles on the body that can be viewed with a dissecting microscope under UV (black light). For individual flies where the dye was not visible, the heads were dissected to reveal dye particles caught in the ptilinum during pupal eclosion. Dye colors used in marking flies included Aurora Pink (used in Florida) and Blaze Orange (used in California) (DayGlo Corporation, Cleveland, OH, USA), and each color was applied at a dose of 2 g per L of pupae.

### Irradiation and insect holding

Prior to irradiation, 1 L of dyed *B*. *dorsalis* bisexual pupae was divided into five lots of 200 mL and sealed in plastic bags (80 cm L x 30 cm W x 20 cm D; length cut to size) by tying them tightly to remove as much air as possible. This procedure was performed 3 h before irradiation to induce hypoxia, which mitigates harmful effects of irradiation on insect quality and physiology [26–28]. Each sealed plastic bag of pupae was placed in one of the five chambers of an RS 2400 Q Sterile Insect X-ray Irradiator (Radsource, Buford, GA, USA), where pupae were irradiated at a target dose of 100 Gy operated at 160 kV and 25 mA. During irradiation, bags of pupae were placed in plastic cylinders with the center point 17 cm from the X-ray source delivering a dose rate of approximately 10 Gy per min and a dose uniformity ratio of 1.6. A sterilization dose of 100 Gy has been implemented in successful SIT programs in mango growing areas of Thailand [29] and Hawaii [30]. Pupae were irradiated at 5 d, i.e., 3 d before emergence [31].

Immediately after irradiation, the pupae were packed in cooler boxes (Styrofoam, internal dimensions 28 cm L x 22 cm W x 19 cm D) with blue ice packs and shipped to California or Florida. For California, the shipment was flown to Los Angeles and then driven to Los Alamitos (ca. 1 h trip) to the headquarters of the California Department of Food and Agriculture (CDFA) Medfly Preventive Release Program. The pupae were shipped from Hilo with layovers in Honolulu, HI and Memphis, TN to the USDA-APHIS-PPQ Sterile Insect Release Facility in Sarasota, FL. Total shipping times were approximately 24 h to Los Alamitos, CA and 42 h to Sarasota, FL.

Upon arrival, pupae were distributed among six eclosion boxes (4800–6400 flies per eclosion box; Rubbermaid® 24 gallon [90.85 liters] action packers [Newell Brands, Atlanta, GA, USA]), modified to have screen-covered openings on the top and sides. A mixture of sugar and protein hydrolysate (3:1 v:v) was placed on each eclosion box as a circular cake (6 cm diameter, 2 cm thick) on the top screen through which the flies could feed. An agar block (15 x 9.6 x 1.5 cm) was provided as a water source. Agar blocks were replaced every 3 d, and food was added as needed. The eclosion boxes were held in rooms maintained at 25 ± 1°C, 55% ± 3% rh, and a 12:12 photoperiod. The flies were held in the eclosion boxes for a total of 12 d and released at 10–12 d of age when sexually mature.

### Insect quality

Standard quality control tests were conducted for flies used in field experiments. These included measurements of adult emergence, flight ability, and 14-d survival for each production batch (i.e., each group of release flies). Tests were conducted at the CDFA Medfly Preventive Release Program in Los Alamitos, CA, and the USDA-APHIS-PPQ Sterile Insect Release Facility in Sarasota, FL, following internationally accepted protocol [32].

One hundred pupae chosen at random were placed in the center of a blackened petri dish. A black PVC tube (10 cm tall, 8 cm diameter) was then placed in the petri dish (80 mm diameter), and the petri dish and tube were placed in a screen cage (52 x 28 x 53 cm). Prior to placement, the inner surface of the tube was coated with unscented talcum powder (except for the bottom 1 cm, which was wiped clean) to prevent the flies from walking out of the tube. Light level was approximately 1,500 lumens. Seven days after emergence, counts were made of fliers, emerged flies that remained in the tube, and un-emerged flies. To measure survival, 50 newly emerged males were placed in a cubical screen cage (30 x 40 x 30 cm) with food (3:1 sugar: yeast hydrolysate mixture) and agar provided *ad libitum*. After 14 d the number of dead flies was counted.

### Preparation, deployment, and density of SPLAT sites

SPLAT-MAT-ME (51% ME; 47% water, waxes, and oils; 2% spinosad (a mixture of spinosyn A and spinosyn D)) was used for each replicate in this study (Dow AgroSciences LLC 62719–592 Indianapolis, IN USA). 60 mL syringes were used to apply 8 mL of the SPLAT-MAT-ME formulation to individual wooden blocks (10.0 x 8.0 x 0.5 cm), which were hung individually in large plastic delta traps (“LPD traps”– 28 x 20 x 15cm) (Scentry Biologicals, Inc., Billings, MT USA) with a hanger that was glued to the wood block. The LPD traps contained removable sticky inserts on the bottom of the traps to capture flies and served as monitoring devices for the attraction and killing of released flies. The LPD traps were deployed, and the SPLAT formulation applied 1d before fly release. LPD traps were hung using a zip tie that was fastened around the tree or pole at approximately 3 m from the ground. For each replicate, the same number (*N* = 30) of LPD traps was deployed uniformly as part of the MAT grid in each density grid. These were a minimum of 100 m from the release transect to permit the flies to disperse prior to encountering a trap.

Additional SPLAT sites were deployed throughout the medium and high-density grids by CDFA and Florida Department of Agriculture and Consumer Services (FDACS) personnel by truck. These additional SPLAT sites were deployed as spot treatments on utility poles and trees that met CA and FL program standards of SPLAT-MAT-ME application and distributed evenly throughout the plots with a minimum distance of 50 m from other SPLAT sites, LPD traps, or the release transects. Forty spot treatments were added in addition to the LPD trap points in the medium treatment, and 120 additional spot treatments were added in the high treatment. The purpose of these additional SPLAT sites was to establish low, medium, and high experimental MAT densities equivalent to 50, 110, and 230 SPLAT sites per km^2^ (120, 280, and 600 per mi^2^), respectively. Thus, the total number of SPLAT sites was 30 for the low (30 LPD, 0 additional sites), 70 for the medium (30 LPD, 40 additional sites), and 150 for the high (30 LPD, 120 additional sites). The number of flies killed at the additional SPLAT sites was not monitored. While the total number of SPLAT sites differed between the three treatments, the same number of LPD traps was used to estimate the total number of flies captured in each grid and allowed comparison between the three densities.

### Release-recapture protocol

Release-recapture timelines followed the same protocol and schedule in all experimental plots in both California and Florida. The LPD traps were deployed, and the SPLAT formulation was applied between 0900 and 1300 1 d before or (in one instance) on the day of fly release. In each plot, flies were released along a centrally located, 150 m transect (i.e., street) from a crate wagon (60.96 x 121.92 x 60.29 cm) (Sandusky Lee CW4824 Littlestown, PA, USA) pulled slowly by a crew member while a second person periodically opened the eclosion box slightly and tapped it to promote flight. An electric leaf blower (typical model used P21012BTL, Ryobi Ltd. Fuchu, Hiroshima, Japan) was used to disperse flies reluctant to leave the box at end of transect or on the wagon and nearby vehicles.

Flies from two eclosion boxes were released in each plot. The estimated number of males released per plot was calculated by the number of pupae (40 pupae per mL), emergence, flight ability and 14 d survival rates from subsamples of pupae. The number of adult flies left in the release boxes because they were dead or otherwise did not leave was counted after the release. The entire dataset including geolocation details is available online [23].

Sticky inserts from the LPD traps were removed 1 and 5 d after release, and the captured flies were counted and submitted to the laboratory for identification. Inserts were replaced at the 1 d post-release check but not at the 5-d check, when traps were removed and properly disposed of following agency guidelines.

### Statistical analysis

A Generalized Linear Model (GLM) with appropriate distributional assumption and link function was fit to the estimated kill relative to number released but not killed. All statistical analyses were conducted in R version 4.1.1 and 4.3.2 [33]. Estimated marginal means (least-squares means) were used as a post hoc test as implemented in the package “emmeans” [34].

## Results

**Fig 1:**
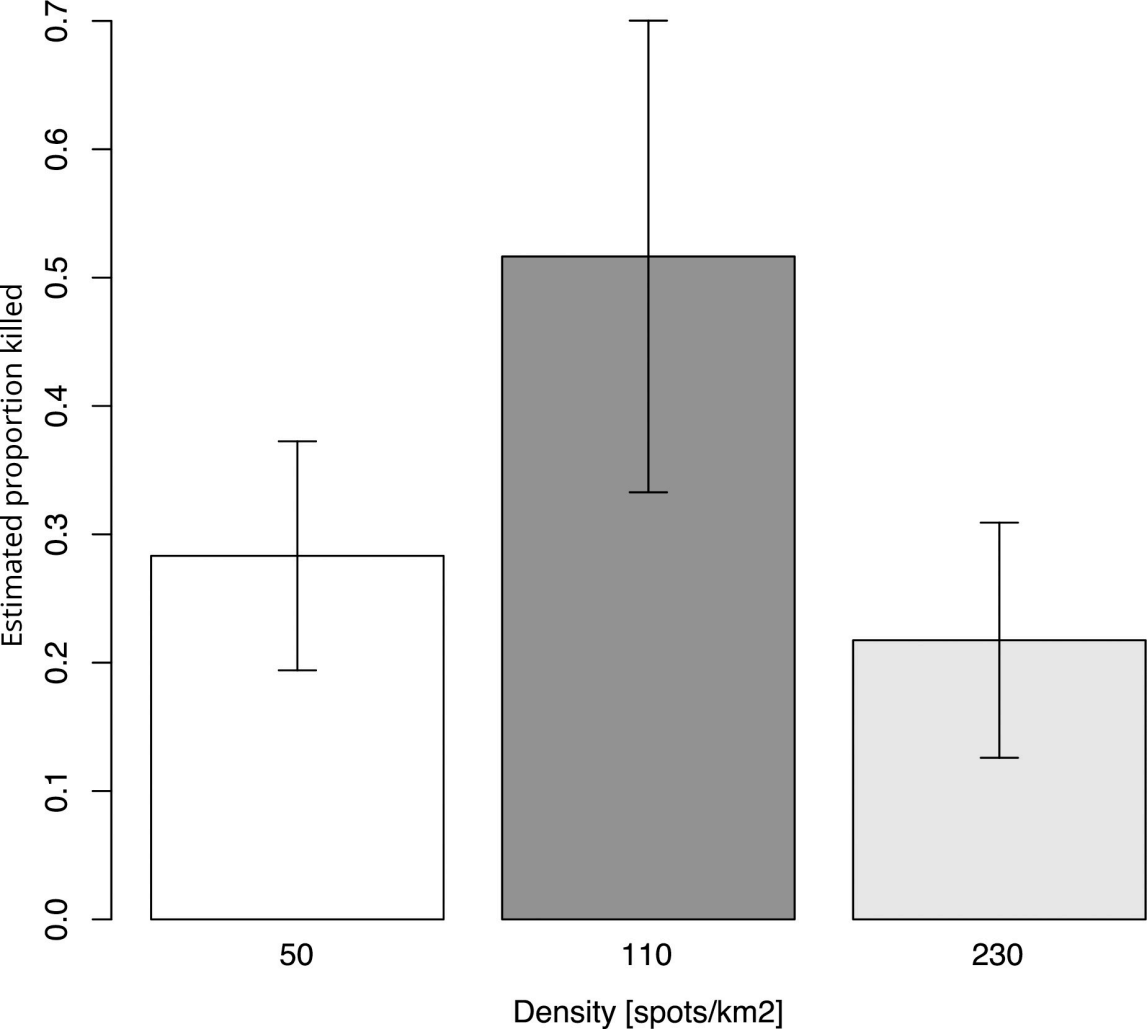
Mean estimated proportion of released male *B*. *dorsalis* killed across trials in California and Florida. Error bars reflect the standard error (SE).

An estimated 4487 to 5270 males were released in each plot for experiments conducted between August 2021 and November 2022 (mean = 4799). Recapture numbers in the 30 LPD traps per plot varied between 87 and 2133 males; no unmarked *B*. *dorsalis* were found in any of the traps. Across trials, the mean number of captures per LPD was 44.2 males (range 13.0–71.1) in the low-density plots (50 spots per km^2^), 34.8 males (range 10.1–64.4) in the middle density (110 spots per km^2^), and 6.95 males (range 2.9–15.8) in the high (“standard”) density plots (230 spots per km^2^). Multiplying the average catch per LPD trap by total number of application spots (LPD + additional sites) yielded the estimated number of marked males killed; this showed that within each trial the middle density was always the treatment with the highest number killed (Table 2). This same result was seen in the average estimated proportion of released males killed across the treatments across all trials, as shown in Fig 1.

**Table 2:** Recapture summary for LPD traps and plot-wide estimates. The average number of estimated males released per experimental plot based on emergence and flight ability data were 4487 in August 2021 (Anaheim, CA), 4588 in September 2021 (Huntington Beach, CA), 4850 in July 2022 (Sarasota-Bradenton 1, FL), and 5270 in November 2022 (Sarasota-Bradenton 2, FL). For data on emergence, flight ability, and remaining number of individuals in each release container, see [23].

Location/Date	Spots/km^2^	Mean LPD	Total LPD	Estim. Kill	Estim. Prop killed
Anaheim/ August 2021	230	4.2	126	630	0.14
	110	45.5	1365	3185	0.71
	50	71.1	2133	2133	0.48
Huntington Beach/ September 2021	230	4.9	146	730	0.16
	110	19.2	575	1342	0.29
	50	31.5	944	944	0.21
Sarasota-Bradenton 1/ July 2022	230	15.8	473	2365	0.49
	110	64.4	1933	4510	0.93
	50	61.1	1833	1833	0.38
Sarasota-Bradenton 2/ November 2022	230	2.9	87	435	0.08
	110	10.1	302	705	0.13
	50	13.0	391	391	0.07

As capture data were not normally distributed a binomial generalized linear model (GLM) was used to test for statistically significant differences between treatments and states where the experiments were conducted (Table 3). The model indicates that both the low and highest (“standard”) treatments had significantly lower kill compared with the middle density treatment. Experiments in Florida had slightly higher kill percentage (average 35% across all treatments) compared with California (average 33% across all treatments); while statistically significant, the magnitude of the difference is negligible.

**Table 3:** Summary of binomial GLM with logit link function. The response variable is estimated number of males killed in all spots (successes) relative to the number released but not killed (failures), and predictors were application density (230, 110, or 50 spots per km^2^) and state (California or Florida). Estimates and significance values for each treatment and for state are relative to 110 spots per km^2^ and California. Null deviance was 18281 on 11 df and residual deviance was 14254 on 8 df. A post hoc test using least-squares means showed that all pairwise comparisons of treatment and state combinations were statistically significantly different from each other except for within treatment comparisons between states (e.g., California 50 was not different from Florida 50; the same was true for the other two treatment levels).

	Estimate	SE	*Z*	*P*
(Intercept)	0.010	0.017	0.56	0.577
Treatment 50	-0.994	0.022	-45.88	<0.0001
Treatment 230	-1.315	0.023	-57.93	<0.0001
Location Florida	0.039	0.018	2.11	0.035

## Discussion

This study supports the hypothesis that the effectiveness of SPLAT-MAT-ME is reduced at the application density that has been considered the standard for the last decades (approximately 230 spots per km^2^). The application density of 110 spots per km^2^ was the most effective treatment for eradicating *B*. *dorsalis*, as measured by the estimated proportion killed. This result is in line with previous research conducted in Hawaii, which also suggests that an application density of 100 or 110 spots per km^2^ is superior to higher application densities [13, 18, 19].

The most likely explanation for reduced captures at the 230 sites per km^2^ density is the ‘MAT-ME saturation hypothesis”, i.e., a large amount of attractant in the environment reduces the ability of individual male flies to locate the source of the odor [18, 19]. If unable to arrive at the SPLAT-MAT-ME application site, then males will not ingest the insecticide, decreasing the effectiveness of the MAT treatment. In the present study the treatment with 110 spots per km^2^ was significantly more effective at capturing and killing male *B*. *dorsalis* than the treatments with 50 and 230 spots per km^2^, suggesting that the efficacy of MAT can be reduced by having too many spots or too few spots per unit area. An excessively high density may create olfactory interference, while a very low density may leave significant areas without olfactory targets within effective range of males.

The underlying principle of MAT-ME saturation is the same as that of “mating disruption”, which is the broadcast application of sex attractant pheromone aimed at reducing the ability of insects to locate mates [35, 36]. The strategy has proven effective for suppressing populations of various moth species, such as *Cydia pomonella* and *Lymantria dispar* [37]. Interference between traps baited with powerful attractants has also been shown to influence captures. For example, Thwaite and Madsen [38] compared two different trap densities for monitoring *C*. *pomonella* in apple orchards and observed that orchards with 1 trap/ha caught an average of 39 codling moths per week, while a higher trapping density of 2.5 traps/ha captured an average of 26.3 moths per week.

The present study was designed to simulate a *B*. *dorsalis* outbreak in areas of the US where a treatment zone would be established around any detection meeting actionable thresholds. Many *B*. *dorsalis* eradications occur in neighborhoods with urban infrastructure, such as shopping malls, recreational parks, and multilane roadways, above and below ground utilities, impacting the spacing and locations of SPLAT-MAT-ME spots. The present study was similarly affected by these limitations, but placement also had to consider spacing of light poles and trees, proximity to schools, shopping malls, and parks, as well as application protocols from CDFA and FDACS, such as those that preclude application of spots over vehicles or on palm trees. While efforts were still made to evenly distribute spots, the previous comparable experiment performed by Manoukis et al. [13] had much more even distribution. Importantly, the same result was evident, with the lower density having a higher estimated proportion killed.

While this experiment aimed to simulate real-world eradication programs, only colony-reared irradiated *B*. *dorsalis* were used in these releases and only estimates of total mortality for entire plots were calculated because not all SPLAT-MAT-ME sites were in traps and flies may have died from causes other than traps, such as predators. Differential emigration from the experimental plots might also have impacted comparability between treatments, but there is no reason to expect this *a priori*.

Colony-reared flies were used in all trials. Tephritids maintained under mass rearing conditions may vary from their wild counterparts with respect to development time, fertility, and survivorship [39, 40]. Additionally, a more recent study conducted by Sim et al. [41] determined that a long-term lab stock of *B*. *dorsalis* had a lower response rate to ME lures in rotating carousel cages compared to a wild-type colony strain. However, since the same colony reared *B*. *dorsalis* were used throughout the present experiment, it is likely that variance introduced by using colony-reared insects impacted all the treatments, so relative results obtained in the present study should apply to wild fruit flies.

MAT has also been used against other tephritids that are not methyl eugenol-responders [12]. The most common non-methyl eugenol MAT employs cuelure (“CL”), a male lure that is effective against *Bactrocera tryoni*, *Zeugodacus cucurbitae*, and other pests of economic concern [42]. Between 1998 and 2000 *Z*. *cucurbitae* was successfully eradicated from the Republic of Naru by means of MAT by deploying a minimum of 300 fiberboards per km^2^ impregnated with CL and the insecticide Fipronil [43]. However, the application density for MAT-CL is usually much higher (400 to 3000 per km^2^ [12]), because cuelure is known to be less attractive to responding males than methyl eugenol [44]. Likely as a result, the recommended application density for MAT-CL is 1600 spots per km^2^. It seems possible that previous MAT efforts using CL might have been negatively impacted by over-application in the same way it might have impacted SPLAT-MAT-ME.

Results from this study have potential implications for reassessing the efficacy of currently prescribed lure densities in other systems, beginning with other methyl eugenol responders. Around 84 species of dacine fruit flies are known responders to ME [45]. Among these, ME response varies across the *Bactrocera dorsalis* sibling species complex, manifested through both dose sensitivity and age of males [46–48]. Differences in responsiveness to ME are discrete enough that they can be used as a behavioral parameter to distinguish among species in the complex [49]. For instance, Schutze et al. [50] synonymized *B*.*invadens*, *B*.*papaya*, and *B*.*philippinensis* with *B*. *dorsalis* through the lens of pheromone composition and sensitivity, concluding that *B*. *carambolae* (carambola fruit fly) remained distinct. *B*. *carambolae* is one-tenth as sensitive to ME and experiences the highest response to it at 28 days post-emergence versus the ten days of *B*. *dorsalis* [48].

MAT programs can potentially target multiple ME responders concurrently, meaning that lure densities must incorporate and blend multiple response factors [51]. While lure density comparisons of *B*. *dorsalis* with species like *B*. *zonata* (peach fruit fly) do not reveal immediate differences, it is notable that *B*. *zonata* requires a greater amount of ME per trap for optimal trap catch so parsing these differences could influence the length of eradication programs [52, 53]. Effective application rates and duration prescribed for one species may not apply to others.

At least two important benefits will result from reducing the application density of SPLAT-MAT-ME against *B*. *dorsalis*. First, a reduction in applied material and labor would reduce eradication program costs. Second, this study as well as previous findings suggest that lower densities are more effective and should improve the effectiveness of the eradication programs used against a highly invasive agricultural pest, thus improving the biosecurity of nations experiencing incursions by *B*. *dorsalis*.
